# Repeatability and agreement of planar lumbar range of motion measured by dynamic fluoroscopy and optical motion capture in an ovine cadaveric model

**DOI:** 10.3389/fbioe.2026.1827364

**Published:** 2026-06-10

**Authors:** Hongtao Li, Guangwei Liu, Changxiao Han, Bochen Peng, Ruiyao Qin, Jiacheng Zheng, Guangyi Yang, Liguo Zhu, Yun Wang, Minshan Feng

**Affiliations:** 1 Department of Spine Surgery, Wangjing Hospital, China Academy of Chinese Medical Sciences, Beijing, China; 2 Beijing Key Laboratory of Digital Intelligence Traditional Chinese Medicine for Preventing and Treating Degenerative Bone and Joint Diseases, Beijing, China; 3 Department of Radiology, Peking Union Medical College Hospital, Chinese Academy of Medical Sciences and Peking Union Medical College, Beijing, China

**Keywords:** dynamic fluoroscopy, lumbar spine, optical motion capture, ovine cadaveric model, range of motion

## Abstract

**Introduction:**

Lumbar range of motion is an important biomechanical parameter for understanding spinal function. However, the repeatability, measurement error, and between-method agreement of dynamic fluoroscopy and optical motion capture remain insufficiently characterized in controlled cadaveric spine testing.

**Methods:**

Planar lumbar kinematics were measured using dynamic fluoroscopy within a dynamic X-ray system (DXRS) and optical motion capture with bone-fixed markers (OMCS) in six fresh-frozen ovine lumbar spine specimens, each comprising a continuous L1-L5 segment, during quasi-static flexion-extension and lateral bending. Three repeated trials were performed for each condition. Repeatability was assessed using the intraclass correlation coefficient (ICC(2,1)), the standard error of measurement (SEM), and the minimum detectable change at the 95% confidence level (MDC95). Between-method agreement was evaluated using a repeated-measures Bland-Altman approach with specimen-level clustering, exploratory proportional-bias assessment, and specimen-level bootstrap confidence intervals (CIs) (10,000 replicates) for the bias and limits of agreement (LoA).

**Results:**

ICC point estimates were generally high within each method across motion conditions and vertebral levels; however, given the modest specimen sample (n = 6), the corresponding 95% CIs were wide and these estimates are reported as preliminary, condition-specific values. OMCS showed numerically smaller SEM and MDC95 than DXRS in this dataset, but specimen-level bootstrap intervals indicated uncertainty around most between-method differences, so these descriptive differences should not be interpreted as evidence of general method superiority. Between-method analysis showed a small positive OMCS-minus-DXRS bias with condition-dependent LoA, and these agreement estimates should likewise be interpreted as preliminary and condition-specific.

**Conclusion:**

In this controlled ovine cadaveric model, both DXRS and bone-fixed OMCS provided repeatable measurements of projected planar lumbar motion. OMCS showed numerically smaller measurement-error indices in this dataset and under the present bone-fixed *ex vivo* ovine configuration; these descriptive, configuration-specific differences do not imply general method superiority, clinical equivalence, or interchangeability with routine human skin-marker motion capture. Cross-device comparisons should account for systematic bias, LoA, and uncertainty related to repeated-measures clustering. The reported error and agreement estimates may help inform method selection and interpretation of longitudinal change in preclinical spine biomechanics studies.

## 1 Introduction

The range of motion (ROM) of the lumbar spine is a key biomechanical parameter essential for understanding spinal function, particularly in the context of injury, rehabilitation, and surgical procedures (Errabity et al., 2023; Barbosa et al., 2023). Accurate measurement of lumbar spine ROM is critical in both clinical and research settings; however, the reliability and measurement error can vary across commonly used ROM assessment approaches and protocols (Johnson and Mulcahey, 2021; McClintock et al., 2024). Such inconsistencies between methods may influence clinical interpretation and biomechanical analyses.

Among the methods used to quantify spinal motion, dynamic X-ray system (DXRS) based imaging and optical motion capture system (OMCS) are particularly relevant to controlled biomechanical testing (Romero-Flores et al., 2024; Breen et al., 2021; Breen and Breen, 2020). Recent validation studies of low-back posture tools and skin-based motion capture systems have emphasized that external or wearable measurement approaches require careful agreement assessment against reference methods, particularly when lumbar kinematics are inferred rather than directly imaged (García-Luna et al., 2024; Frey et al., 2025). DXRS imaging enables direct visualization of skeletal structures and can quantify continuous intervertebral kinematics under standardized motion tasks (Breen et al., 2021; Breen and Breen, 2020). Nevertheless, fluoroscopy uses ionizing radiation and therefore requires attention to radiation protection and dose considerations, which can constrain repeated assessments (Fisher et al., 2022; Gr et al., 2026).

OMCS provides a non-ionizing alternative and is well suited for repeated assessments, but it infers skeletal motion from externally tracked markers rather than directly imaging bone (Romero-Flores et al., 2024). Marker placement error and soft-tissue artifact can introduce substantial error when estimating lumbar vertebral kinematics (Severijns et al., 2021; John et al., 2024), motivating concern about agreement when OMCS-derived measures are compared against X-ray-based approaches (Frey et al., 2025; Wang et al., 2021).

In experimental biomechanics, OMCS can be implemented using either skin-mounted markers or markers rigidly coupled to the skeleton. Rigid coupling (e.g., vertebra-attached rigid bodies/markers) is used in cadaveric testing to minimize soft-tissue artifact and establish a best-case tracking benchmark (Orbach et al., 2023; Page et al., 2022). Currently, comparative studies on the quantification of lumbar ROM and planar kinematics using DXRS and OMCS technologies are still relatively limited (Frey et al., 2025; Wang et al., 2021). Animal models, particularly ovine specimens, provide a valuable translational platform due to documented anatomical/biomechanical similarities to humans and their suitability for controlled *ex vivo* lumbar biomechanics testing (Easley et al., 2008; Bonilla et al., 2025).

Therefore, this study aimed to evaluate the within-method repeatability and between-method agreement of projected planar lumbar ROM measured by DXRS and bone-fixed OMCS using fresh-frozen ovine lumbar spine segments. Six continuous ovine lumbar spine segments (L1-L5) were tested during flexion-extension and lateral bending, with three repeated trials performed for each condition. By using a harmonized planar angle definition and explicit plane alignment between systems, this study compared method-specific measurement error and agreement using ICC, SEM, MDC95, and repeated-measures Bland-Altman analyses in a controlled preclinical biomechanics setting.

## 2 Materials and methods

### 2.1 Ethics statement

The animal study was approved by the Ethics Committee of Peking Union Medical College Hospital, Chinese Academy of Medical Sciences and Peking Union Medical College (approval No. I24PJ0512). All procedures were performed in accordance with institutional guidelines and relevant national regulations.

### 2.2 Specimen preparation

**FIGURE 1:**
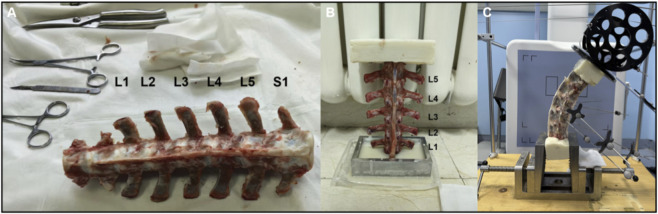
Surgical handling and fixation of ovine specimens. **(A)** Surgical anatomy of the ovine lumbar spine; **(B)** Rigid fixation of the ovine lumbar spine model; **(C)** Fixation of the ovine lumbar spine model on a horizontal tabletop. Vertebral levels L1-L5 are labeled in **(A,B)**.

Six fresh-frozen ovine lumbar spine specimens were obtained from a commercial supplier, Yancheng Baihan Biotechnology Co., Ltd. (Yancheng, China), including continuous L1-L5 segments with intervertebral discs, facet joints, and major ligamentous structures preserved. Surrounding musculature and adipose tissue were carefully removed while maintaining the integrity of the osseoligamentous structures to preserve passive mechanical integrity (Figure 1A). Specimens were kept moist throughout preparation and testing using 0.9% saline to prevent dehydration, and were allowed to equilibrate to room temperature prior to experimentation. To rigidly secure the specimen ends for mounting, both ends of each lumbar segment were embedded (potted) in rectangular aluminum molds using a self-curing dental acrylic system (dental powder and self-curing liquid; Feiying, China). The acrylic was prepared according to the manufacturer’s instructions and poured into the molds to encapsulate the superior and inferior ends, forming rigid potting blocks after curing. Testing was performed after the acrylic had fully cured (Figure 1B). The potting blocks were then used to clamp and fix the specimen to the custom loading apparatus, ensuring stable boundary conditions during quasi-static flexion-extension and lateral bending tests (Figure 1C).

### 2.3 Marker placement and vertebral rigid-body representation

To enable stable kinematic tracking with optical motion capture, Kirschner wires (K-wires) were rigidly inserted into selected vertebrae, and reflective markers were mounted on the extracorporeal ends of the wires to ensure rigid coupling between markers and the underlying bone. Within each L1-L5 segment, a total of nine reflective markers were used, including three markers attached to L1, three to L3, and three to L4. For each instrumented vertebra, the three markers were arranged in a non-collinear configuration to define a vertebral rigid body for orientation estimation. These instrumented vertebrae were selected to provide representative measurements across the tested segment while maintaining sufficient marker visibility and minimizing marker crowding during synchronized optical and fluoroscopic acquisition.

### 2.4 Dynamic X-ray system

**FIGURE 2:**
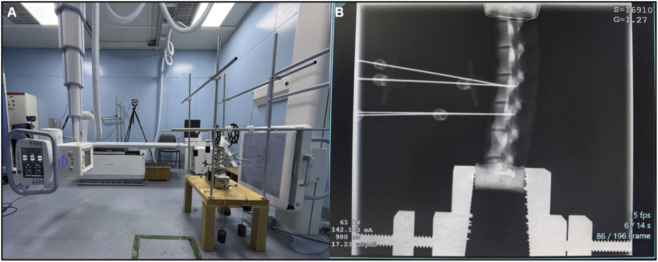
Dynamic X-ray System. **(A)** Dynamic X-ray system device; **(B)** DI-X1 image diagnostic processing software used for fluoroscopic image analysis.

Dynamic fluoroscopy was acquired using a dynamic X-ray system (AeroDR C80; Konica Minolta Medical Technology (Shanghai) Co., Ltd., Shanghai, China) (Figure 2A). Dynamic fluoroscopy was used to quantify vertebral motion in the sagittal plane (for flexion-extension) and the coronal plane (for lateral bending). Fluoroscopy images were acquired at 6 frames per second (fps). Acquisition parameters were 65 kVp and 140 mA with a 5 m exposure time per pulse. Added filtration (1 mm Al + 0.5 mm Cu) was applied, and the source-to-image distance (SID) was 1.8 m. Image acquisition was performed automatically by the DXRS system. For kinematic analysis, vertebral motion was quantified from the planar orientation of the superior vertebral endplate at stable endpoint frames. The endplate line was generated using software-assisted image analysis and then visually checked by the operator. Frames were accepted when the endplate boundary was clearly visible, the fitted line was anatomically consistent with the visible superior endplate contour, and no obvious tracking or fitting failure was present. If the software-assisted line was inconsistent with the visible endplate contour, the frame was rechecked and the endplate line was corrected before endpoint angles were extracted for ROM computation (Figure 2B).

### 2.5 Optical motion capture system

**FIGURE 3:**
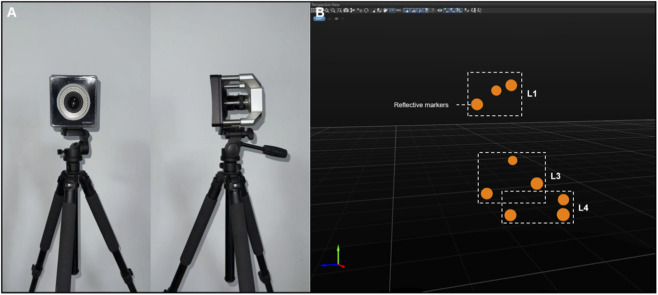
Optical motion capture system. **(A)** Optical motion capture device; **(B)** Motive software used for optical motion capture processing. The figure labels the orange dots as reflective markers mounted on the extracorporeal ends of K-wires. Three non-collinear markers defined each tracked vertebral rigid body and were used for OMCS tracking of L1, L3, and L4.

Optical motion capture was performed using an OptiTrack system (NaturalPoint, Inc., United States) with six Prime 41 cameras (4.1-megapixel resolution) (Figure 3A). Marker trajectories were sampled at 120 Hz. Vertebral orientations were reconstructed from the marker-defined rigid-body representations. A calibration object with known geometry was used to establish the experimental reference axes and to align the OMCS coordinate frame with the DXRS imaging plane. OMCS-derived vertebral orientations were transformed into the DXRS plane reference frame and projected onto the DXRS imaging plane. The same planar angle variable was then computed relative to the base horizontal axis so that OMCS and DXRS used a harmonized planar angle definition (Figure 3B).

### 2.6 Motion protocol and mechanical loading

**FIGURE 4:**
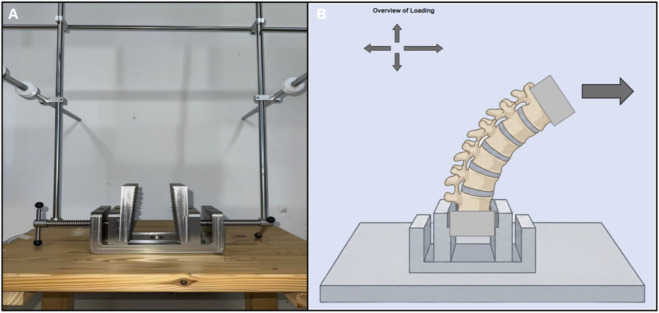
Mechanical loading of specimens. **(A)** A custom mechanical loading apparatus. **(B)** Schematic loading-direction overview.

Specimens were mounted on a custom mechanical loading apparatus, with the inferior end rigidly fixed to the experimental base (Figure 4A). Passive lumbar motion was generated by applying external loads to the superior end using a pulley-lever system. Both flexion-extension and lateral bending motions were produced under symmetric bilateral loading, with two 1-kg weights applied in a balanced configuration at the loading end. Loads were gradually applied until the specimen reached a stable static extreme position and were then reversed to complete an endpoint-to-endpoint cycle along the same loading path (Figure 4B). Loading direction was adjusted to produce motion primarily within the sagittal plane for flexion-extension and within the coronal plane for lateral bending. The specimens were not mechanically constrained to pure two-dimensional motion; therefore, coupled axial rotation and other out-of-plane motions could occur, particularly during lateral bending. All tests were conducted under quasi-static conditions, focusing on endpoint-based kinematics rather than load-displacement relationships. No neutral reference posture was defined. Each specimen underwent three repeated trials for each motion condition.

### 2.7 Data processing and outcome measures

OMCS marker trajectories were processed in Motive (OptiTrack, NaturalPoint, Inc., United States), where marker labeling and rigid-body reconstruction were performed. The resulting trajectories and orientations were exported using the software default processing and export pipeline, and no additional user-defined filtering parameters such as filter type, cutoff, order, or phase characteristics were specified during post-processing. Exported trajectories therefore reflect software default settings and no additional filtering beyond those defaults was applied by the user. Vertebral orientations were then projected onto the DXRS imaging plane prior to planar angle computation. The primary outcome measure was projected planar ROM, defined as the absolute difference between planar endpoint angles measured at the two extreme positions of each motion cycle for flexion-extension and lateral bending.

### 2.8 Statistical analysis

All analyses were performed in R version 4.0.3 (R Foundation for Statistical Computing, Vienna, Austria). Analyses were performed separately by motion condition and vertebral level, with specimen treated as the experimental unit. Repeated trials were not treated as independent specimens. Within-method repeatability was assessed separately for DXRS and OMCS using ICC(2,1), defined as a two-way random-effects, absolute-agreement, single-measure intraclass correlation coefficient. SEM was estimated from the residual error variance of the corresponding repeated-measures model, and MDC95 was calculated as 1.96 × √2 × SEM. Because SEM and MDC95 are measurement-error indices rather than formal tests of method superiority, between-method differences in these values were evaluated only as an exploratory sensitivity analysis. For each motion-by-level condition, 10,000 specimen-level cluster bootstrap replicates were generated by resampling specimens with replacement while retaining all repeated trials and paired DXRS/OMCS observations within each selected specimen. ΔSEM was defined as SEM_DXRS −SEM_OMCS, and ΔMDC95 was defined as MDC95_DXRS − MDC95_OMCS. Percentile 95% confidence intervals (CIs) were calculated for both ΔSEM and ΔMDC95. Between-method agreement was assessed using repeated-measures Bland-Altman analysis. The paired difference was defined as OMCS − DXRS, and the paired mean was defined as (OMCS + DXRS)/2. Paired differences were fitted using a linear mixed-effects model with a random intercept for specimen, equivalent to difference ∼1 + (1 | specimen). The fixed intercept was used as the mean bias, and the specimen-level and residual variance components were used to calculate the 95% limits of agreement (LoA). Specimen-level cluster bootstrap resampling with 10,000 replicates was used to estimate percentile 95% CI for the bias and LoA. Proportional bias was assessed as an exploratory secondary check using difference ∼ paired mean + (1 | specimen). Linear mixed-effects models were fitted using the lme4 package in R. Pearson’s r was additionally reported as a descriptive measure of linear association between DXRS and OMCS, not as a measure of agreement or interchangeability. Because repeated measurements were clustered within specimens, inferential P values for Pearson correlation were not calculated or interpreted. Continuous variables are presented as mean ± standard deviation.

## 3 Results

Six specimens were tested under flexion-extension and lateral bending. Planar ROM derived from endpoint angles at L1, L3, and L4 were measured using DXRS and OMCS. Each condition was repeated three times (trials 1–3), yielding 18 observations per method × motion × level cell (6 specimens × 3 trials) and 18 paired observations per motion × level cell for between-method comparisons. Trial-wise mean ± SD and pooled (overall) mean ± SD are summarized in Table 1. Overall (pooled) mean values were similar between OMCS and DXRS for both flexion-extension and lateral bending at L1, L3, and L4.

**TABLE 1:** Trial-wise and overall mean ± SD (DXRS vs. OMCS).

Method	Motion	Level	Trial 1 (degree)	Trial 2 (degree)	Trial 3 (degree)	Overall (degree)
DXRS	Flexion-extension	L1	54.8 ± 9.5	56.2 ± 9.2	56.5 ± 5.6	55.8 ± 7.8
DXRS	Flexion-extension	L3	49.2 ± 11.0	49.2 ± 8.7	49.3 ± 8.2	49.2 ± 8.8
DXRS	Flexion-extension	L4	42.2 ± 9.7	40.3 ± 9.6	40.9 ± 10.4	41.1 ± 9.3
DXRS	Lateral bending	L1	66.8 ± 12.7	65.0 ± 11.4	65.1 ± 10.9	65.6 ± 11.0
DXRS	Lateral bending	L3	50.8 ± 7.2	48.9 ± 6.8	49.8 ± 7.3	49.8 ± 6.7
DXRS	Lateral bending	L4	41.9 ± 8.2	40.9 ± 6.8	39.9 ± 8.2	40.9 ± 7.3
OMCS	Flexion-extension	L1	55.2 ± 9.3	57.0 ± 8.7	57.2 ± 5.8	56.5 ± 7.7
OMCS	Flexion-extension	L3	50.6 ± 9.9	50.5 ± 9.2	50.0 ± 8.4	50.3 ± 8.6
OMCS	Flexion-extension	L4	42.6 ± 9.8	41.1 ± 9.2	42.9 ± 9.2	42.2 ± 8.8
OMCS	Lateral bending	L1	67.3 ± 13.0	65.8 ± 11.7	66.2 ± 10.8	66.5 ± 11.2
OMCS	Lateral bending	L3	51.3 ± 6.9	50.1 ± 7.2	50.5 ± 7.2	50.6 ± 6.7
OMCS	Lateral bending	L4	42.4 ± 8.1	41.9 ± 7.6	40.8 ± 7.7	41.7 ± 7.3

ICC point estimates were high across conditions (OMCS ICC(2,1): 0.811–0.969; DXRS: 0.796–0.949), but several 95% CIs were wide because of the modest specimen sample, so the ICC values are presented as preliminary, condition-specific estimates rather than as definitive method-comparison values (Table 2). Across all motion-by-level conditions, OMCS showed numerically smaller SEM and MDC95 than DXRS. These between-method differences are descriptive only; the specimen-level cluster bootstrap sensitivity analysis (Supplementary Table S1) indicates the uncertainty around ΔSEM and ΔMDC95, and these results should not be interpreted as evidence of general OMCS superiority.

**TABLE 2:** ICC, ICC 95% CI, SEM, and MDC95.

Method	Motion	Level	ICC (2,1)	ICC 95% CI	SEM (degree)	MDC95 (degree)
OMCS	Flexion-extension	L1	0.811	0.061–0.941	3.6	10.1
OMCS	Flexion-extension	L3	0.947	0.748–0.981	2.3	6.4
OMCS	Flexion-extension	L4	0.943	0.603–0.969	2.2	6.2
OMCS	Lateral bending	L1	0.969	0.608–0.983	2.1	5.9
OMCS	Lateral bending	L3	0.934	0.341–0.957	1.9	5.2
OMCS	Lateral bending	L4	0.962	0.315–0.982	1.4	3.9
DXRS	Flexion-extension	L1	0.796	0.150–0.911	3.9	10.9
DXRS	Flexion-extension	L3	0.876	0.501–0.945	3.6	9.9
DXRS	Flexion-extension	L4	0.928	0.469–0.975	2.7	7.5
DXRS	Lateral bending	L1	0.949	0.437–0.978	2.7	7.4
DXRS	Lateral bending	L3	0.918	0.514–0.967	2.0	5.5
DXRS	Lateral bending	L4	0.929	0.378–0.970	2.0	5.6

ICC(2,1) denotes a two-way random-effects, absolute-agreement, single-measure intraclass correlation coefficient. SEM, and MDC95 are measurement-error indices and were not interpreted as formal tests of method superiority.

**FIGURE 5:**
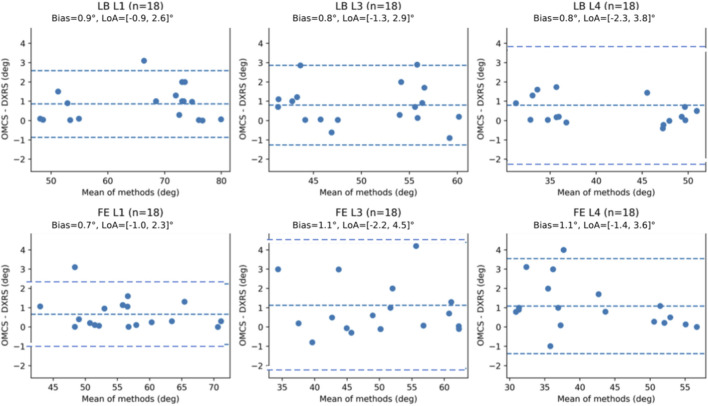
Repeated-measures Bland-Altman agreement between OMCS and DXRS. Each panel shows paired ROM measurements for one motion-by-level condition (n = 18 paired observations; 6 specimens × 3 repeated trials). The x-axis shows the paired mean of the two methods and the y-axis shows the difference (OMCS-DXRS, degree). The central dashed line indicates mean bias and the upper/lower dashed lines indicate the 95% LoA. Bias and LoA were estimated using a specimen-level repeated-measures structure; bootstrap CIs are reported in Supplementary Table S2.

**FIGURE 6:**
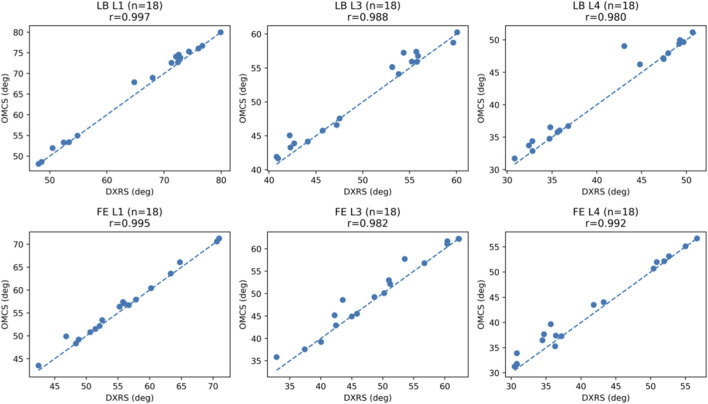
Scatter plots and Pearson correlation between DXRS and OMCS. Scatter plots of OMCS versus DXRS for flexion-extension and lateral bending at L1, L3, and L4 (n = 18 per panel; 6 specimens × 3 repeated trials). The dashed line represents the line of identity (y = x). Pearson’s r is reported in each panel title to describe linear association between methods.

Between-method agreement is shown in Table 3; Figure 5. Repeated-measures Bland-Altman analysis showed a small positive systematic bias for OMCS minus DXRS across all conditions, indicating that OMCS reported, on average, slightly higher planar ROM values than DXRS in this dataset. The 95% LoA showed that individual paired observations could differ by several degrees, with condition-dependent width; given the modest sample, both the bias and the LoA (and their specimen-level bootstrap CIs, Supplementary Table S2) are reported as preliminary, condition-specific agreement estimates rather than as definitive cross-method interchangeability bounds. The exploratory proportional-bias assessment suggested a possible condition-specific mean-dependent pattern for flexion-extension at L3, whereas the remaining conditions did not show a stable proportional-bias pattern. Pearson’s r indicated strong linear association between methods across conditions but was interpreted only as trend consistency, not as agreement or interchangeability (Table 4; Figure 6).

**TABLE 3:** Bland-Altman bias and LoA.

Motion	Level	n (paired)	Bias (degree)	LoA lower (degree)	LoA upper (degree)
Flexion-extension	L1	18	0.7	−1.0	2.3
Flexion-extension	L3	18	1.1	−2.2	4.5
Flexion-extension	L4	18	1.1	−1.4	3.6
Lateral bending	L1	18	0.9	−0.9	2.6
Lateral bending	L3	18	0.8	−1.3	2.9
Lateral bending	L4	18	0.8	−2.3	3.8

**TABLE 4:** Pearson correlation.

Motion	Level	n (paired)	Pearson r	P value (descriptive)
Flexion-extension	L1	18	0.995	-
Flexion-extension	L3	18	0.982	-
Flexion-extension	L4	18	0.992	-
Lateral bending	L1	18	0.997	-
Lateral bending	L3	18	0.988	-
Lateral bending	L4	18	0.980	-

Pearson correlation was reported only as a descriptive measure of linear association, not as a formal test of agreement or interchangeability. Because repeated measurements were clustered within specimens, inferential P values were not calculated or interpreted.

## 4 Discussion

This study compared within-method repeatability and between-method agreement of projected planar lumbar kinematics measured by dynamic fluoroscopy within a DXRS and by bone-fixed OMCS in a fresh-frozen ovine lumbar spine model under quasi-static flexion-extension and lateral bending. Using three repeated trials per condition, both modalities showed high ICC point estimates across levels and motions, but the precision of these estimates was limited by the modest specimen sample and should be viewed as preliminary and condition-specific. OMCS showed numerically smaller SEM and MDC95 across the tested conditions; consistent with the framing used throughout the manuscript, these differences are treated as descriptive, configuration-specific measurement-error findings under the present bone-fixed *ex vivo* ovine model, and not as evidence of statistical or general method superiority. Between-method comparisons demonstrated a small positive systematic bias with OMCS minus DXRS and condition-dependent LoA, indicating that the two systems track similar magnitudes at the group level but are not strictly interchangeable for individual measurements without considering bias and error bounds.

An important methodological implication of these findings is the availability of quantitative error thresholds that can guide interpretation of longitudinal change. While ICC values were generally high for both systems, ICC alone does not specify the magnitude of change required to exceed measurement noise. SEM and MDC95 provide more actionable quantities for interpreting pre-post differences and follow-up changes (Deane et al., 2020). In the present dataset, OMCS showed numerically smaller SEM and MDC95 than DXRS across all motion-by-level conditions; however, the exploratory specimen-level bootstrap analysis was used to describe uncertainty around these between-method error differences, not to test general method superiority, with only flexion-extension at L3 showing a condition-specific bootstrap interval that strictly excluded zero (Supplementary Table S1). Therefore, these findings should be interpreted as preliminary, exploratory, condition-specific observations under the present bone-fixed *ex vivo* ovine cadaveric configuration, not as evidence that OMCS has generally lower measurement error or that the two methods are clinically interchangeable. Changes below the relevant MDC95 remain more likely attributable to measurement variability than to true kinematic alteration.

Between-method agreement further clarifies the extent to which values can be compared across systems. Repeated-measures Bland-Altman analysis revealed a modest positive bias across all conditions, indicating that OMCS tended to report slightly higher projected planar values than DXRS. Although mean bias was small, LoA suggested that individual paired observations could differ by several degrees, with agreement varying by motion and level. When agreement is assessed with repeated observations per experimental unit, clustered or mixed-effects approaches are needed to account for within-specimen dependence and avoid overstating precision (Parker et al., 2020). This distinction is methodologically important because similarity of pooled means and strong correlation do not imply interchangeability. The exploratory proportional-bias assessment suggested a possible condition-specific mean-dependent pattern only for flexion-extension at L3, and this should be interpreted cautiously because only six specimens were available. If cross-system comparisons are unavoidable, the present bias estimates and LoA provide preliminary, condition-specific uncertainty bounds under the tested experimental setting, and should not be taken as proof of method interchangeability or as transferable reference values for routine clinical motion analysis.

The observed bias and condition-dependent LoA likely reflect modality-specific measurement mechanisms despite a unified planar angle definition. DXRS estimates endplate orientation directly from fluoroscopic images, whereas OMCS derives vertebral orientation from marker-defined rigid bodies and then projects that orientation into the DXRS imaging plane using calibration-based alignment. In addition, nominally planar loading may include coupled axial rotation and other out-of-plane motions, particularly during lateral bending. This coupling between lateral bending and axial rotation is consistent with prior *in vitro* evidence that spinal segments can exhibit coupled three-dimensional motion during lateral bending (Orbach et al., 2023). Such three-dimensional motion can alter the projected planar angle measured by each system and may contribute to broader LoA in some motion-level combinations. Therefore, the present outcome should be understood as projected planar endpoint ROM rather than complete three-dimensional vertebral kinematics. Because DXRS captures endplate orientation directly from the imaged bony contour while OMCS reconstructs orientation from a rigid-body marker triad and then projects it onto the DXRS plane, the two methods may differ in their sensitivity to coupled out-of-plane motion when reduced to a single planar angle, which could partly account for the condition-dependent LoA observed here.

From an application perspective, DXRS has a distinct advantage when direct visualization of osseous motion is required, because it can directly visualize skeletal structures and quantify bone motion with high anatomical face validity. Fluoroscopy-based approaches have been used to derive continuous lumbar intervertebral motion and can demonstrate reliable motion patterns under standardized protocols *in vivo*, supporting their role as a bone-based reference in spine kinematics research (Breen et al., 2021). However, routine use is constrained by ionizing radiation exposure, limiting frequent repeated measurements in longitudinal follow-up (Fisher et al., 2022; Gr et al., 2026; Van Ngoc Ty et al., 2024). OMCS offers a non-ionizing alternative with high sampling frequency and may be useful for repeated assessment in controlled laboratory environments. Nevertheless, the OMCS configuration used in this study relied on K-wire bone-fixed markers, which represent a best-case tracking scenario with minimized soft-tissue artifact. This configuration is not equivalent to routine human skin-marker OMCS, where marker placement error, optical occlusion, and soft-tissue artifact can substantially affect lumbar kinematic estimates (Xi et al., 2022). Accordingly, the present findings should not be generalized to clinical skin-marker motion capture without further validation.

A methodological strength of this work is the explicit harmonization of variable definitions and coordinate frames. By aligning OMCS to the DXRS imaging plane using a calibration object and computing an identical projected planar angle relative to the same base axis, the comparison minimizes disagreement attributable to inconsistent coordinate conventions. Furthermore, the use of repeated trials enabled reporting of SEM and MDC95 alongside ICC, while the revised specimen-level bootstrap analyses provide a transparent assessment of uncertainty around descriptive error differences and Bland-Altman agreement estimates.

## 5 Limitations and future directions

Several limitations should be acknowledged. First, the sample size was modest (n = 6 specimens), and although repeated trials were included, the specimen remained the experimental unit; therefore, the estimated SEM, MDC95, bias, LoA, and bootstrap CIs should be interpreted as preliminary, condition-specific method-comparison estimates rather than definitive population-level or cross-setting reference values (Parker et al., 2020). Second, the present outcome was projected planar endpoint ROM rather than complete three-dimensional vertebral kinematics. Because the specimens were not mechanically constrained to pure two-dimensional motion, coupled axial rotation and other out-of-plane motions may have occurred, particularly during lateral bending, but were not directly quantified (Orbach et al., 2023). Third, both systems have method-specific sources of uncertainty. DXRS relied on software-assisted endplate tracing with operator visual checking, whereas OMCS tracked only selected vertebral levels using bone-fixed markers; rater-related uncertainty in vertebral landmark or endplate identification and marker-configuration limitations may therefore influence measurement precision (Jang et al., 2023). Finally, the cadaveric ovine model and K-wire bone-fixed OMCS configuration represent a controlled best-case experimental setting and should not be directly generalized to intact humans or routine skin-marker motion capture, where neuromuscular control, physiologic loading, marker placement error, optical occlusion, and soft-tissue artifact may substantially affect lumbar kinematic estimates (Severijns et al., 2021; John et al., 2024; Xi et al., 2022). Future studies should include larger cohorts, full three-dimensional kinematic analysis, dynamic tasks, and direct validation of skin-marker OMCS in human participants.

## 6 Conclusion

In this controlled *ex vivo* ovine cadaveric model with n = 6 specimens, DXRS and bone-fixed OMCS both produced repeatable measurements of projected planar lumbar ROM under a harmonized planar angle definition and explicit plane-alignment procedure. The numerically smaller SEM and MDC95 observed for OMCS, the ICC point estimates and 95% CIs, and the Bland-Altman bias and 95% LoA are presented as preliminary, condition-specific method-comparison estimates under the present bone-fixed *ex vivo* configuration, and do not imply general OMCS superiority, clinical equivalence, or interchangeability with routine human skin-marker motion capture. The reported error and agreement estimates may inform method selection and the interpretation of longitudinal change in controlled preclinical spine biomechanics studies, pending replication in larger cohorts.

## Data Availability

The raw data supporting the conclusions of this article will be made available by the authors, without undue reservation.
