# Supplementary material for: Repeatability and agreement of planar lumbar range of motion measured by dynamic fluoroscopy and optical motion capture in an ovine cadaveric model
Source: Front Bioeng Biotechnol. 2026 Jun 10;14:1827364. doi: 10.3389/fbioe.2026.1827364 (PMC13291058; doi:10.3389/fbioe.2026.1827364)
Supplement: Supplementary file 1 [file Table1.docx]

**Supplementary Table S1. Exploratory specimen-level cluster bootstrap comparison of SEM and MDC95 between DXRS and OMCS.**

Legend: Delta values were calculated as DXRS minus OMCS. Positive values indicate numerically smaller SEM/MDC95 for OMCS. Bootstrap confidence intervals were estimated using specimen-level cluster resampling with 10,000 replicates. This is an exploratory sensitivity analysis, not a primary superiority test. For lateral bending L1, the lower bounds of the bootstrap 95% CIs for both ΔSEM and ΔMDC95 fell exactly at 0.00 (boundary). We conservatively interpret these CIs as crossing zero and therefore not providing evidence of a non-zero between-method error difference.

| Motion | Level | SEM_DXRS | SEM_OMCS | Delta SEM | 95% CI for Delta SEM | MDC95_DXRS | MDC95_OMCS | Delta MDC95 | 95% CI for Delta MDC95 | Bootstrap valid n | Interpretation |
| --- | --- | --- | --- | --- | --- | --- | --- | --- | --- | --- | --- |
| Flexion-extension | L1 | 3.93 | 3.64 | 0.3 | -0.18 to 0.77 | 10.9 | 10.08 | 0.82 | -0.49 to 2.13 | 10000 | CI crosses zero; descriptive difference only |
| Flexion-extension | L3 | 3.58 | 2.29 | 1.29 | 0.12 to 1.66 | 9.92 | 6.35 | 3.56 | 0.34 to 4.59 | 10000 | CI does not cross zero; exploratory evidence of smaller error under this condition |
| Flexion-extension | L4 | 2.69 | 2.23 | 0.45 | -0.27 to 0.83 | 7.45 | 6.19 | 1.26 | -0.76 to 2.30 | 10000 | CI crosses zero; descriptive difference only |
| Lateral bending | L1 | 2.68 | 2.12 | 0.55 | 0.00 to 0.99 | 7.42 | 5.89 | 1.53 | 0.00 to 2.74 | 10000 | CI crosses zero; descriptive difference only |
| Lateral bending | L3 | 2.0 | 1.86 | 0.14 | -0.33 to 0.55 | 5.55 | 5.16 | 0.39 | -0.92 to 1.52 | 10000 | CI crosses zero; descriptive difference only |
| Lateral bending | L4 | 2.01 | 1.4 | 0.6 | -0.36 to 1.29 | 5.56 | 3.88 | 1.68 | -1.00 to 3.59 | 10000 | CI crosses zero; descriptive difference only |

**Supplementary Table S2. Repeated-measures Bland-Altman agreement analysis with exploratory proportional-bias assessment.**

Legend: Bias was defined as OMCS minus DXRS. Limits of agreement were estimated using specimen-level repeated-measures variance components. Confidence intervals and proportional-bias slope intervals were obtained by specimen-level cluster bootstrap with 10,000 replicates. Proportional bias was assessed as an exploratory secondary check. “Boundary variance estimate” indicates whether the estimated specimen-level random-intercept variance component was approximately zero in that condition. In these cases, the repeated-measures variance-component estimate was close to the conventional Bland-Altman estimate, and results should be interpreted as descriptive agreement estimates rather than as evidence of absence of specimen-level variability. In such cases the repeated-measures limits of agreement collapse toward the conventional (non-clustered) Bland-Altman limits.

| Motion | Level | n pairs | Bias | 95% CI for bias | Lower LoA | 95% CI for lower LoA | Upper LoA | 95% CI for upper LoA | Proportional-bias slope | 95% CI for slope | Boundary variance estimate | Bootstrap valid n | Interpretation |
| --- | --- | --- | --- | --- | --- | --- | --- | --- | --- | --- | --- | --- | --- |
| Flexion-extension | L1 | 18 | 0.66 | 0.39 to 1.00 | -0.96 | -1.35 to -0.51 | 2.28 | 1.33 to 3.30 | -0.061 | -0.305 to 0.045 | Yes | 10000 | Slope CI crosses zero; no stable mean-dependent pattern in this exploratory check |
| Flexion-extension | L3 | 18 | 1.13 | 0.55 to 1.83 | -2.24 | -2.85 to -1.67 | 4.50 | 2.84 to 5.87 | -0.454 | -0.664 to -0.191 | Yes | 10000 | Slope CI does not cross zero; exploratory condition-specific mean-dependent pattern |
| Flexion-extension | L4 | 18 | 1.09 | 0.57 to 1.64 | -1.38 | -2.03 to -0.74 | 3.55 | 2.08 to 4.81 | -0.177 | -0.386 to 0.106 | No | 10000 | Slope CI crosses zero; no stable mean-dependent pattern in this exploratory check |
| Lateral bending | L1 | 18 | 0.86 | 0.54 to 1.19 | -0.91 | -1.35 to -0.40 | 2.63 | 1.88 to 3.43 | -0.239 | -0.397 to 0.019 | Yes | 10000 | Slope CI crosses zero; no stable mean-dependent pattern in this exploratory check |
| Lateral bending | L3 | 18 | 0.80 | 0.31 to 1.30 | -1.28 | -1.61 to -0.80 | 2.88 | 1.90 to 3.64 | -0.127 | -0.332 to 0.274 | No | 10000 | Slope CI crosses zero; no stable mean-dependent pattern in this exploratory check |
| Lateral bending | L4 | 18 | 0.78 | 0.34 to 1.36 | -2.25 | -3.60 to -0.75 | 3.81 | 1.58 to 6.20 | -0.37 | -1.207 to 0.082 | Yes | 10000 | Slope CI crosses zero; no stable mean-dependent pattern in this exploratory check |

Note: Wider bootstrap confidence intervals (e.g., lateral bending L4 LoA upper 95% CI width >4.5°) reflect greater uncertainty in agreement estimates and should be considered when applying these LoA values for cross-device comparisons. In the four conditions where the specimen-level variance estimate was at the boundary (FE L1, FE L3, LB L1, LB L4), the estimated specimen-level variance component for paired differences was at the boundary (≈ 0), so the repeated-measures and conventional Bland-Altman limits of agreement are numerically similar; this indicates that, for these conditions, the between-method bias was relatively consistent across the six specimens. In the remaining two conditions (FE L4, LB L3), a non-zero specimen-level variance component was retained, meaning that between-method bias varied across specimens within those conditions. These patterns should be interpreted in light of the small specimen sample (n = 6).
